# An update to the Milk Allergy in Primary Care guideline

**DOI:** 10.1186/s13601-019-0281-8

**Published:** 2019-08-12

**Authors:** Adam Fox, Trevor Brown, Joanne Walsh, Carina Venter, Rosan Meyer, Anna Nowak-Wegrzyn, Michael Levin, Hannah Spawls, Jolene Beatson, Marie-Therese Lovis, Mario C. Vieira, David Fleischer

**Affiliations:** 1 Department of Paediatric Allergy, Guys and St Thomas’ Hospitals NHS Foundation Trust, London, UK; 2 Paediatric Allergy, Ulster Hospital, Belfast, BT16 1RH Northern Ireland; 3 Gurney Surgery, Castle Partnership, 70 Fishergate, Norwich, NR3 1SE UK; 4 Section of Allergy and Immunology, University of Colorado Denver School of Medicine, Children’s Hospital Colorado, 13123 East 16th Avenue, Anschutz Medical Campus, Box B518, Aurora, CO 80045 USA; 5 Department Paediatrics, Imperial College, London, London, W2 1NY UK; 6 Jaffe Food Allergy Institute, Icahn School of Medicine at Mount Sinai, New York, NY 10029 USA; 7 Division of Paediatric Allergy and Asthma, Red Cross War Memorial Children’s Hospital, University of Cape Town, Room 516, ICH Building, Cape Town, South Africa; 8 Cow’s Milk Protein Allergy Support Group, 5 Cypress Grove, School Aycliffe, Co Durham DL5 6GP UK; 9 The Wall House Surgery, Yorke Road, Reigate, RH2 9HG UK; 10 Center for Pediatric Gastroenterology, Hospital Pequeno Príncipe, Curitiba, Brazil; 11 Department of Pediatrics, Pontifical Catholic University of Paraná, Curitiba, Brazil

**Keywords:** Cow’s milk allergy, Guidelines, Food allergy, Diagnosis, MAP, iMAP

## Abstract

The Milk Allergy in Primary (MAP) Care guideline was first published in 2013 in this journal. MAP aimed to provide simple and accessible algorithms for UK clinicians in primary care, detailing all the steps between initial presentation, through diagnosis, management and tolerance development. Despite its UK focus, it soon became clear that MAP was being accessed internationally and thus an updated International Milk Allergy in Primary Care (iMAP) guideline was published in 2017. Both guidelines used existing international consensus guidelines to develop accessible algorithms accompanied by patient information leaflets. In 2018, the guidelines were criticised for 3 distinct reasons: promoting the overdiagnosis of cow’s milk allergy (CMA), negatively impacting breastfeeding and the possibility of industry influence on the guidelines. The authors address these criticisms using available evidence and, in the context of this and in consultation with patient groups, members of the General Practice Infant Feeding Network and other infant feeding healthcare leads, have collaboratively produced updated algorithms and an information leaflet to support breastfeeding. We believe iMAP is now closer to its original aim of facilitating early and accurate diagnosis of CMA, whilst minimising, as far as possible, any concerns around overdiagnosis or a risk to breastfeeding rates. We continue to welcome open and constructive engagement about how best to achieve these aims to provide evidence-based, practical guidelines for the primary care practitioner.

**Electronic supplementary material:**

The online version of this article (10.1186/s13601-019-0281-8) contains supplementary material, which is available to authorized users.

The Milk Allergy in Primary Care guideline was first published in 2013 in this journal by five authors [1], four of whom had been involved in the development group of the UK National Institute for Health and Care Excellence (NICE) 2011 clinical guideline on the ‘Diagnosis and assessment of food allergy in children and young people in primary care and community settings’ [2]. The driver for the development of primary care focussed cow’s milk allergy (CMA) guidance was the limitations of the scope of the NICE guideline, which did not include management of food allergy, nor any specific detail relating to the challenges of identifying and diagnosing milk allergy, which can present with diverse clinical symptoms, due to either an underlying IgE or a non-IgE mediated mechanism. There was good evidence that delay in diagnosis was a common problem for patients, particularly in infants with less severe manifestations of non-IgE mediated milk allergy, and this resulted in a significant [3], unnecessary morbidity and anxiety. This was commonly reported by patients to the authors in their own clinical practice.

MAP aimed to provide simple and accessible algorithms for clinicians in primary care, detailing all the steps between initial presentation, through diagnosis and management as well as later follow up to assess for tolerance development, which is almost always seen in early childhood for those children with non IgE mediated milk allergy [1]. In healthcare environments where there is minimal specialist allergy provision, it remains important that mild-moderate non-IgE mediated CMA can be diagnosed accurately and promptly in the primary care setting where these infants are most likely to present. It was considered that a tool which focussed on the UK primary care setting was therefore needed, but soon it became clear that MAP was being accessed internationally, with almost 89,000 accesses to date. This initial guideline was therefore updated in 2017 as International Milk Allergy in Primary Care (iMAP) [4]. Seven further authors, representing five other continents, helped to modify the algorithms such that they could act as a template suitable for local adaptation in different international healthcare settings.

Neither MAP nor iMAP claimed to be the result of new evidence reviews, but instead used the existing international consensus guidelines (such as DRACMA, NIAID, NICE, EAACI, ESPGHAN, BSACI) [5–8] to develop easy to use algorithms accompanied by a range of patient information leaflets to help support healthcare professionals seeing infants with symptoms which may represent CMA. The diagnostic and management steps in MAP/iMAP were entirely consistent with these guidelines, and the list of symptoms of mild to moderate non-IgE mediated CMA largely repeated the symptoms listed from other guidelines to illustrate the range of potential presentations. In practice, whilst the algorithms were widely distributed, these were often used without the important context provided by the accompanying article. Any algorithm has to balance the accessibility brought by brevity with the reduced clinical context, potentially leading to the less nuanced decision-making that this may bring.

## A Time for Reflection

In 2018, the iMAP/MAP guidelines were criticised for 3 distinct reasons which will be considered in turn and could be broadly generalised to any of the available CMA guidelines [9]. It is extremely important to consider how the concerns raised could constructively inform changes to the current iMAP guideline in a way that mitigates any potential risks to best practice. In anticipation of the need for the algorithms to evolve over time, these had been hosted on the Allergy UK (UK National allergy charity) website for ease of access and also for ongoing adaptation of a ‘live’ version.

The first criticism raised is that iMAP promotes overdiagnosis of CMA by suggesting that a large range of common and non-specific symptoms could represent mild to moderate non-IgE mediated CMA. Dietary changes, such as mothers being advised to exclude milk from their diet as part of a diagnostic elimination diet trial or the prescription of hypoallergenic formulas, could thus be happening unnecessarily due to an over-perception of disease. The evidence cited for this was a dramatic increase in prescriptions for hypoallergenic milk formulas over a 10-year period from 2006 (7 years prior to publication of MAP) to 2016, coupled with an absence of evidence of a meaningful increase in the prevalence of CMA during this period. However, the evidence cited with regards to a lack of change in the prevalence of milk allergy related to the same cohort of children at 2 different time points, rather than 2 distinct groups of infants [9]. There was no consideration that there may be an increase in true prevalence, better recognition of previously undiagnosed CMA, or simply more hypoallergenic formula prescribed because soya formula, which these babies may previously have taken, was no longer considered suitable under 6 months of age, and an alternative was needed. It has also been reported that children with mild to moderate non-IgE mediated CMA may have been wrongly labelled with lactose intolerance, with this happening less commonly as awareness of CMA improved, resulting in more prescriptions for hypoallergenic formula and less for lactose-free formula. Critics also questioned whether the passage of ß-lactoglobulin, a cow’s milk protein, through the breastmilk of nursing mothers, can occur in high enough concentration to cause symptoms in the milk-allergic infant. However, data does support the existence of a CMA in breastfed infants from a prospective observational study of 1749 infants, with 2.2% (n = 39) fulfilling the criteria of CMA; of these milk allergic infants, 9 (23%) presented with symptoms whilst exclusively breastfed, representing 0.5% of the entire sample [10]. In addition, the ß-lactoglobulin levels in breastmilk from mothers consuming cow’s milk, which ranges between 0.5–150 μg/L, is similar to the residue in extensively hydrolysed formulas where reactions have been well-described [11–15].

**Fig. 1:**
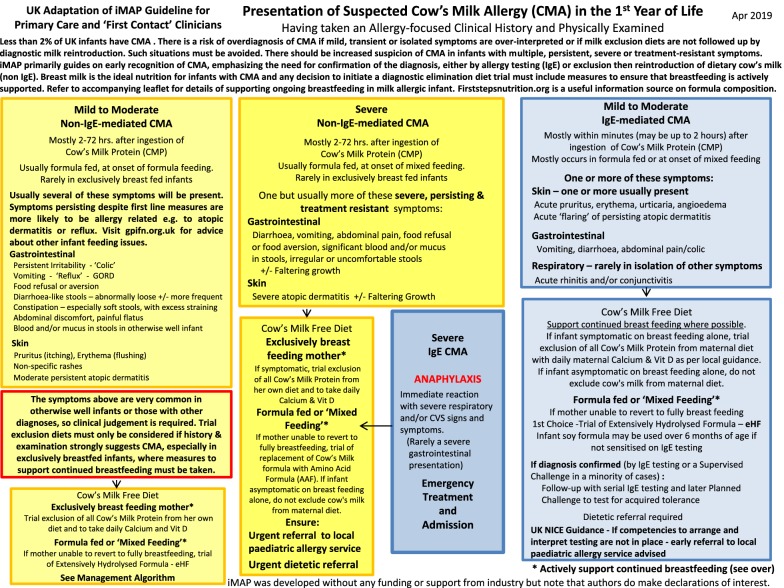
Presentation of suspected cow’s milk allergy (CMA) in the 1st year of Life

**Fig. 2:**
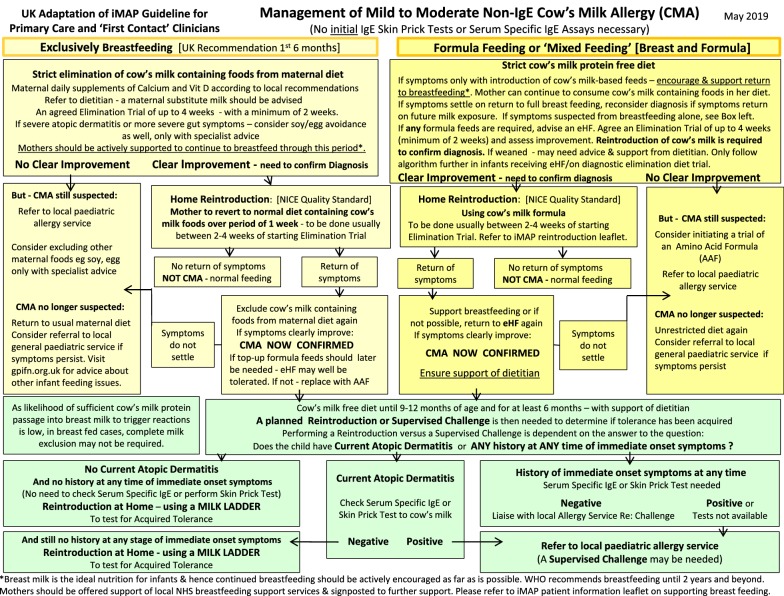
Management of mild to moderate non-IgE cow’s milk allergy (CMA)

Whilst we do not agree that either MAP or other guidelines have driven the increased prescriptions of hypoallergenic formulas, the authors acknowledge that the symptoms of mild to moderate non-IgE mediated CMA do overlap significantly with a large number of completely well infants, and whilst it is not within the gift of physicians to change the symptoms of the diseases that we try to recognise, it is extremely important that patients are protected from unnecessary diagnostic elimination diet trials. In the absence of a reliable test or widely used, validated scoring questionnaire for symptoms, the guideline therefore needs to better highlight the importance of not over-interpreting minor symptoms, especially in exclusively breastfed infants, where the likelihood of symptoms being related to milk is much lower than in formula fed infants [10]. The guideline algorithm (Figs. 1 and 2) have thus been revised to highlight the current prevalence of CMA, the importance of using clinical judgement when interpreting symptoms, weighting those that are multiple, persistent, severe or treatment resistant, and drawing direct attention to the danger of overdiagnosis in this context.

In response to the suggestion that guidelines are driving an increase in formula prescription rates, it should be considered that there are no reliable prospective data the authors are aware of to assess if there has been any change of CMA prevalence over the period in question, or any other time period. Some of the most robust, challenge-based data from the Europrevall study estimate a UK prevalence of CMA of 1.28% at 2 years of age, of which approximately half is due to non-IgE mediated allergy [16]. If there has been an increase in maternal perception of CMA and subsequent overdiagnosis by doctors during the period from 2006 to 2016, it would be unlikely to have been driven by MAP, which was not published until 2013. Indeed, data on the amount spent by the UK NHS on extensively hydrolysed and amino acid formula can be seen to rise sharply from 2003, 10 years prior to publication of MAP [17]. The increase in UK prescriptions for specialist formula also predates the publication of the first CMA focussed guidelines by 4 years [18]. Furthermore, these earlier guidelines had neither a UK nor primary care focus, did not include any practical algorithms, and were not well known outside the speciality setting; therefore, it is also very unlikely that these could have meaningfully contributed to any systematic, erroneous overdiagnosis.

The issue of over-perception of allergy is very well established and was demonstrated by Venter et al. [19] in a study of infants born in 2001–2002 on the Isle of Wight, when over 33% of parents reported a food allergy in their child, even though the overwhelming majority were later shown not to have one based upon oral food challenges. In this cohort, over the course of the first 3 years of life, 2.8% of children were diagnosed with CMA based on DBPCFC, with the majority suffering from mild to moderate non-IgE mediated CMA. The authors do not believe that there is any evidence to support the assertion that CMA guidelines have led to overdiagnosis of CMA, and furthermore believe that the data demonstrating rapidly increasing sales of specialist formula many years prior to the publication of the guidelines, strongly evidence the lack of a causal relationship. The publication of iMAP, which has seen much broader uptake than the initial MAP guideline (reported as used by 16.2% of a sample of 266 GPs, significantly less than local GP guidelines or GP notebook, but more than the 4.1% using MAP) [20], in 2017, has been followed by a complete plateau in NHS spending of hypoallergenic formula, and in fact, data from IQVIA show a decline in sales on these formula from £64.54 m in 2017 to £64.51 m in 2018, the year following the release of iMAP. Therefore, the data further refutes the suggestion that MAP or iMAP are contributing to increased hypoallergenic formula sales.

The authors believe that effective education and high-quality guidance would work against misdiagnosis (both over and underdiagnosis), and consequently avoid unnecessary morbidity or inappropriate prescriptions [21], by providing information for parents and their doctors, rather than incorrect diagnoses being made through a lack of either knowledge or the correct information being given to families. It is also important to note that in 2003, the UK Department of Health issued guidance relating to the use of soy-based infant formulas [22], at the time the mainstay for infants with CMA, advising that they no longer be used in infants under 6 months because of concerns about phytoestrogen levels. This would provide a plausible explanation for a marked uptake in alternative hypoallergenic formulas without requiring any meaningful increase in the number of infants being diagnosed, and is wholly supported by electronic Prescribing Analysis and Cost (ePACT) data showing a marked decline in prescriptions for soy-based formula for the period 2003–2008, falling from 17,114 prescriptions nationally in October 2003 to 8369 prescriptions in September 2008 [17]. It is also noted that the period from 2003–2016 was a time of exponential growth in social media, where it is well established that parents seek out their healthcare advice, much of which comes from uninformed sources, and is very likely to have driven parental concerns around possible food allergy significantly more than a healthcare professional’s guideline. Both of these factors coincide with the increase of sales of hypoallergenic formulas seen and may offer a plausible explanation for at least a large component of them, that is consistent with the data available.

It is also important to note that one of the most likely causes of overdiagnosis is misclassification of patients as being allergic, due to failure to conduct a re-challenge to milk after a brief exclusion [23]. Without confirming that symptoms return on reintroduction of milk following a diagnostic elimination diet trial, only a presumptive diagnosis of CMA can be made, and there is a risk that infants whose symptoms in fact resolved spontaneously, and not as a result of milk exclusion, will be wrongly labelled as milk-allergic. We believe that a guideline which emphasizes the need for re-challenge to cow’s milk, before a diagnosis can be made, is vital to protect against this risk, given the lack of a reliable test.

A further criticism relates to the risk that the guideline may negatively impact breastfeeding rates. Published data on breastfeeding rates in the UK indicated that only 1% of mothers still exclusively breastfeed at 6 months of age, as per World Health Organization Guidelines [24]. Although 81% of mothers start to breastfeed, within one week, half have already started infant formula [25]. The causes of the poor breastfeeding rates in the UK are multi-factorial and clearly have social, cultural and political components, with the lack of healthcare professional support for breastfeeding well described. As highlighted in the UNICEF call to action and the Lancet Breastfeeding Series [26], there are clear public health indications for supporting breastfeeding to be every clinician’s responsibility and we were keen to review any area where the iMAP may be felt to undermine this. Breastmilk remains the ideal source of nutrition for the cow’s milk-allergic infant. There are 2 ways that MAP/iMAP has been criticised with regards to breastfeeding. The first is that by raising the possibility that benign symptoms may be related to CMA, this may encourage mothers to consider excluding milk from their diet unnecessarily, with some concern that being on a restrictive diet could contribute to a decision to stop breastfeeding. The other criticism is that the guideline does not emphasize the importance of breastfeeding enough, especially in the context of infants whose symptoms develop when milk-based infant formula is first introduced. There may be a lost opportunity here to actively encourage mothers to return to breastfeeding, rather than simply exchange the milk-based formula for a hypoallergenic one and simple amendments to the guideline can help to mitigate this risk. We have listened to these concerns, recognised that the importance of breastfeeding could be better highlighted and taken measures in the updated algorithms to do this, actively encouraging guideline users to support mothers to continue breastfeeding where at all possible, and to only consider hypoallergenic formulas if this is not possible. We have also developed a new iMAP patient information leaflet which signposts some of the different resources that can specifically support the breastfeeding diet (Additional file 1). We have consulted both parent groups and breastfeeding support groups with specific reference to both the updated algorithms and the new patient information leaflet.

The final criticism relates to the possibility of industry influence on the guidelines. This has been compounded by the widespread dissemination of the MAP/iMAP algorithms as part of branded promotional material by infant formula manufacturers. We wish to highlight that MAP/iMAP guidelines were published in an open access publication to ensure free access to this educational resource and thus industry branding of the guideline did not require the agreement of the authors, nor was it within their control. Prior to MAP, most CMA guidelines were developed directly with industry funding. MAP and iMAP received no industry funding at all at any stage of development. However, all of the original authors have declared interests relating to work, predominantly around research funding, educational grants or consultancy, with infant formula manufacturers, similar to other national and international clinical guidelines [1, 4]. Whilst these declarations comply with ethical obligations around transparency, there remains a potential risk of unconscious bias, especially when the guidelines relate to the products from which the companies involved are profiting. Such potential for bias may be mitigated through the peer review process, but one possible further method of managing this concern is to widen the circle of those involved in developing the guidance. To this end, the current iteration of the MAP guideline has received patient input from members of a large, online CMA community, Cow’s Milk Protein Allergy Support, members of the General Practice Infant Feeding Network and other infant feeding healthcare leads, none of whom has any industry ties. Their advice, received without payment, has led to this version of the guideline hopefully demonstrating a renewed commitment to helping clinicians to support continued breastfeeding and recognising the public health priority of health care workers doing so.

In developing the new iteration of MAP/iMAP, we have attempted to understand the criticisms and address them directly. This guidance will never be perfect for everyone, but we believe it is now closer to its original aim of facilitating early and accurate diagnosis of CMA, whilst minimising, as far as possible, any concerns around overdiagnosis or a risk to breastfeeding rates. We welcome an open and constructive engagement about how best to achieve these aims to provide evidence-based, practical guidelines for the practitioner and where further changes may bring us closer to this. We believe that a collaborative approach to this to be in the best interest of our patients, which is something that we all share (Additional file 1).

## Additional files

**Additional file 1.** Initial fact sheet for infants with symptoms of a possible mild to moderate non-IgE mediated allergy whilst being exclusively or partly breastfed.

## Data Availability

Data sharing not applicable to this article as no datasets were generated or analysed during the current study.

## Abbreviations

MAP: Milk Allergy in Primary Care
CMA: cow’s milk allergy
UK: United Kingdom
US: United States
EAACI: European Academy of Allergy, Asthma and Clinical Immunology
NICE: National Institute of Health and Care Excellence
BSACI: British Society for Allergy and Clinical Immunology
ESPGHAN: European Society for Paediatric Gastroenterology Hepatology and Nutrition
GPs: general practitioners
iMAP: International version: Milk Allergy in Primary Care
eHFs: extensively hydrolysed formulas
AAF: amino acid-based formula
IgE: immunoglobulin E
DRACMA: Diagnosis and Rationale for Action Against Cow's Milk Allergy
NIAID: National Institute of Allergy and Infectious Diseases
ePACT: Electronic Prescribing Analysis and Cost
DBPCFC: double-blind, placebo-controlled food challenge
NHS: National Health Service
UNICEF: United Nations Children's Fund
