# Supplementary material for: An update to the Milk Allergy in Primary Care guideline
Source: Clin Transl Allergy. 2019 Aug 12;9:40. doi: 10.1186/s13601-019-0281-8 (PMC6689885; doi:10.1186/s13601-019-0281-8)
Supplement: Supplementary file 1 — Additional file 1. Initial fact sheet for infants with symptoms of a possible mild to moderate non-IgE mediated allergy whilst being exclusively or partly breastfed. [file 13601_2019_281_MOESM1_ESM.pdf]

The iMAP Milk Allergy Guideline

– initial fact sheet for infants with symptoms of a possible mild to moderate non-IgE mediated allergy whilst being exclusively or partly breastfed

iMAP GUIDELINE

You have been given this factsheet because your baby may be showing mild-to-moderate symptoms of a delayed type of allergic reaction (see fact sheet on symptoms). This may be due to the cow's milk proteins transferred through breast milk or, the symptoms have occurred only when you first introduced cow's milk containing foods, such as a top-up infant formula, whilst breastfeeding. As breastmilk remains the best source of nutrition for infants, the iMAP Milk Allergy Guidelines want to support mothers to continue breastfeeding and avoid the use of a hypoallergenic formula.

If your baby's symptoms only began when you introduced a top-up infant formula, then the ideal way forward would be to revert back to breastfeeding only (see below for details of support available for ongoing breastfeeding). If the symptoms resolve when you go back to just breastfeeding but return on later re-introduction of milk in food or formula, then the diagnosis of a mild-to-moderate non-IgE mediated allergy should be discussed and revisited with your healthcare professional at that point.

IMPORTANT FACTS:

- Mild-to-moderate non-IgE mediated cow's milk allergy in exclusively breastfed babies is rare and is not a reason to stop breastfeeding. Many of the symptoms are also very common in otherwise well infants or those with other diagnoses. You should only be cutting milk out of your diet if your doctor feels that there is a convincing history to suggest a cow's milk allergy.
- Delayed mild - to - moderate (or non-IgE mediated) cow's milk allergy can only be diagnosed through an elimination diet (to confirm that symptoms improve), followed by the reintroduction of cow's milk into the breastfeeding mother's diet (to confirm a return of symptoms).
- The diagnosis of a mild-to-moderate cow's milk allergy is not complete in an infant showing symptoms when solely breastfed without the re-introduction of cow's milk into the mother's diet and a consequential re-occurrence of symptoms. Any improvement on milk exclusion could be co-incidental.

ADVANTAGES OF CONTINUING TO BREASTFEED:

Breast milk is the best nutrition for babies including those with cow's milk allergy. Breastfeeding contributes to the health of both the mother and infant in the short and longer term. Importantly, breast feeding may play a role in the prevention of allergic diseases. www.thelancet.com/series/breastfeeding

HEALTH BENEFITS FOR THE INFANT	HEALTH BENEFITS FOR THE MOTHER
Breastfeeding **reduces** the infant's risk of developing:	Breastfeeding **reduces** the mother's risk developing:
Infections, including in particular ear infections	Breast cancer
Diarrhoea and vomiting	Ovarian cancer
Sudden infant death syndrome	Osteoporosis
Childhood leukaemia	Cardiovascular disease
Type 2 diabetes	Obesity
Obesity	
Cardiovascular disease in adulthood	
Benefits the infant's developing immune system	
Supports the development of the infant's gastrointestinal tract & healthy gut bacteria	

See also: https://www.bda.uk.com/foodfacts/breastfeeding

ELIMINATING COW'S MILK FROM THE MATERNAL DIET:

Beta-lactoglobulin is a cow's milk protein that can be found in the breast milk of cow's milk consuming mothers and can elicit a non-IgE mediated reaction. Similarly, other allergenic proteins, including soya and egg can be found in breastmilk in sufficient amounts to lead to reactions, however less frequently than cow's milk. It is therefore important not to avoid these unless specifically advised to do so. Breastfeeding mothers also need to know where to get nutritional, medical and emotional support to ensure the continuation of breastfeeding, for the benefit of both mother and child.

When a diagnosis of mild-to-moderate non-IgE mediated allergy is suspected, you will be advised to eliminate cow's milk and its derivatives from your diet. It is important that whilst you do this, you take a vitamin D supplement and ensure sufficient calcium intake, which may also be taken as a supplement. (This should be discussed with your healthcare professional). There are plenty of tasty, calcium enriched milk (and soya) free alternatives (including milk free cheese, yoghurts and even chocolates) that ideally a dietitian will be able to advise you on. You can also obtain useful information from the British Dietetic Association https://www.bda.uk.com/foodfacts/milk_allergy.

If this is a mild-to-moderate non-IgE mediated allergy, symptoms should improve within 2-4 weeks after completely eliminating cow's milk. Ensure to alert your doctor/dietitian if you are experiencing adverse effects on your health as a result of this diet, including weight loss.

If your baby's symptoms improve when you are eliminating cow's milk from your own diet, you need to reintroduce it to your own diet to confirm the diagnosis. If this step is not completed, there is a risk of mis-diagnosis, as any improvements in symptoms may not have been related to the milk exclusion. This should be done by reintroducing cow's milk and cow's milk containing foods into your own diet over a 1-week period, in amounts that you would normally consume (see the MAP information sheet on diagnostic milk reintroduction). If there is a clear re-occurrence of previous gastrointestinal and/or skin symptoms early on, the maternal reintroduction of cow's milk can be stopped before you have completed the full week trial, as this is strongly suggestive of a cow's milk allergy.

ASSOCIATIONS THAT CAN FURTHER SUPPORT BREASTFEEDING:

There are many sources of support to help you to continue breastfeeding for as long as you want to.
Some of these are listed below.

1. Local NHS Breastfeeding support service (details in your baby's red book) - a good first stop.
2. Breastfeeding support telephone lines:
   - National Breastfeeding Helpline – 0300 100 0212.
   - Association of Breastfeeding Mothers – 0300 330 5453.
   - National Childbirth Trust (NCT) – 0300 330 0700.
3. La Leche League (0345 120 2918) provides very useful information on breastfeeding on their website, you can find a local group that may be able to also support you. www.laleche.org.uk
4. The Breastfeeding Network provides support for breastfeeding and have drop in centres and provide telephone support. https://www.breastfeedingnetwork.org.uk/breastfeeding-support/
5. Breastfeeding support group for children with cow's milk allergy https://www.facebook.com/groups CMPASforBreastFeeding/.
6. The NHS also has support websites - https://www.nhs.uk/conditions/pregnancy-and-baby/benefits-breastfeeding/ and https://www.nhs.uk/conditions/pregnancy-and-baby/problems-breastfeeding/
7. GPs can access information on www.gpifn.org.uk to support you through your breastfeeding journey.
8. First Steps Nutrition - https://www.firststepsnutrition.org/eating-well-infants-new-mums
9. Start4Life Breastfeeding Friend – an NHS supported Facebook group that offers advice day or night.
